# Cell death mechanisms in heat stroke and sepsis: ZBP1 and caspase-11 as molecular sensors driving the MLKL/GSDMD death execution axis

**DOI:** 10.3389/fcell.2026.1778368

**Published:** 2026-02-11

**Authors:** Xiao-Jie Qin, Cai-Shen Luo, Qi-Qi Li, Biao-Chao Zhong, Qian-Tong Wei, Chun-Hui Zeng, Ke Yang

**Affiliations:** 1 School of Pharmacy, Guangxi University of Chinese Medicine, Nanning, China; 2 Department of Pharmacy, Faculty of Chinese Medicine Science Guangxi University of Chinese Medicine, Nanning, China; 3 Guangxi Engineering Research Center for High-Value Utilization of Guangxi-Produced Authentic medicinal Herbs, Guangxi University of Chinese Medicine, Nanning, China; 4 University Engineering Research Center of Characteristic Traditional Chinese Medicine and Ethnomedicine, Guangxi University of Chinese Medicine, Nanning, China

**Keywords:** heat stroke, necroptosis, programmed cell death, pyroptosis, sepsis

## Abstract

Heat stroke and sepsis are a pair of acute critical illnesses with distinct underlying causes yet remarkably similar final outcomes. Heat stroke arises from high-temperature environments, disrupting the body’s heat production and dissipation balance; sepsis stems from infection, triggering an uncontrollable inflammatory storm. Both conditions carry extremely high mortality rates and poor prognoses, causing near-total damage to organs and tissues throughout the body. Existing clinical treatments cannot fully reverse the damage inflicted by these diseases. Recent studies have identified necroptosis mediated by the Z-DNA-binding protein 1 (ZBP1) - receptor interaction protein kinase 3 (RIPK3) - mixed lineage kinase-like protein (MLKL) signaling pathway and pyroptosis mediated by the cysteine-aspartic acid proteases-11 (caspase-11) - Gasdermin D (GSDMD) pathway as key mechanisms in heat stroke and sepsis, respectively. Therefore, this review synthesizes recent research findings to analyze the convergent cellular programmed death mechanisms of these two distinct conditions from the perspectives of molecular sensors (a probe for disease triggers) and cell death effectors: ZBP1 senses heat stress, while caspase-11 responds to LPS signaling, initiating downstream membrane-breaching mechanisms executed by MLKL and GSDMD. These processes converge and jointly drive organ damage. The shared pathological outcomes of these distinct diseases suggest that developing broad-spectrum inhibitors targeting their common downstream cell death pathways may represent a novel therapeutic direction.

## 1 Introduction

In recent years, driven by the environmental factor of global warming, the frequency of heatwave events worldwide has continued to rise. This has significantly increased the thermal stress on the human body, leading to an upward trend in the incidence of heat-related illnesses (Li et al., 2018; Schramm et al., 2021; Nori-Sarma et al., 2022). Heat stroke is a severe form of heat illness that occurs when the body is exposed to high temperatures and humidity for an extended period, leading to impaired thermoregulation and excessive heat accumulation. This causes the body’s core temperature to rise above 40 °C (104 °F) (Bouchama et al., 2022). The mortality rate among hospitalized patients with heat stroke reaches as high as 63% (Ebi et al., 2021). Elderly patients with underlying medical conditions face an even higher risk of death and have a poorer prognosis (Ji et al., 2024; Bouchama and Knochel, 2002). Clinically, sepsis—a common ICU condition—shares numerous similarities with heat stroke. Sepsis is defined as a life-threatening organ dysfunction caused by a dysregulated host response to infection. Triggering factors include various acute and chronic diseases such as diabetes, acquired immunodeficiency syndrome, trauma, and bacterial infections (Angus et al., 2001; Singer et al., 2016). According to statistics, there were approximately 49.8 million cases of sepsis worldwide in 2017, with 11 million patients dying from the condition (Fleischmann et al., 2016). Heat stroke and sepsis have fundamentally different causes. The former stems from environmental heat exposure, while the latter arises from systemic infection. Yet both exhibit remarkably similar pathophysiological phenomena and clinical phenotypes. As the disease progresses, both conditions trigger uncontrolled cytokine storms and systemic inflammatory response syndrome (SIRS), disrupt the body’s coagulation function leading to disseminated intravascular coagulation (DIC), and cause multi-organ dysfunction or even failure (Epstein and Yanovich, 2019; Kilic et al., 2009; Rittirsch et al., 2008). For these two acute critical illnesses, traditional treatment approaches fail to yield satisfactory outcomes. The lack of targeted interventions addressing core pathogenic mechanisms makes it difficult to improve disease prognosis.

With deepening research into the molecular mechanisms of disease, programmed cell death has been demonstrated to play a pivotal role in both heat stroke and sepsis. Given the similar terminal outcomes of the two diseases, this phenomenon suggests that their lethal processes are not solely triggered by upstream causes but are more dependent on downstream effect mechanisms. Specifically, programmed cell death mechanisms may be the underlying cause for the convergent pathological endpoints observed in these two distinct diseases. Current therapeutic approaches primarily target upstream etiological interventions, such as cooling strategies for heat stroke and anti-infective strategies for sepsis (Bouchama et al., 2007; Giamarellos-Bourboulis et al., 2024). While these interventions can partially alleviate etiological stimuli, they struggle to interrupt the already activated common lethal pathways—a potential reason for suboptimal treatment outcomes. Building upon this foundation, this paper will transcend the traditional “trigger-symptom” framework. By comparing and integrating heat stroke and sepsis at the molecular sensor - death executor-organ damage levels within cellular programmed cell death pathways, it will reveal their dynamic network characteristics of “separate initiation-cross-talk-terminal convergence.” This aims to provide novel insights and perspectives for treatment strategies targeting these two highly lethal critical illnesses.

## 2 Molecular sensors vs. death actuators: comparison, integration, and signal convergence

**FIGURE 1:**
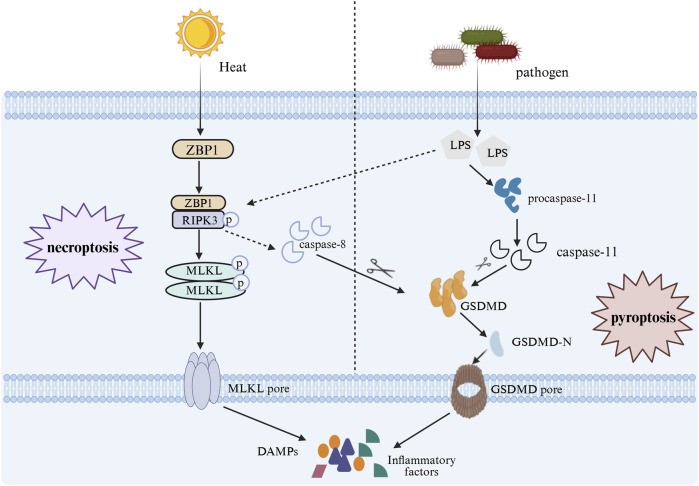
In heat stroke, heat stress activates the ZBP1-RIPK3-MLKL signaling pathway to induce necroptosis in cells, in sepsis, LPS activates caspase-11 to cleave GSDMD, triggering pyroptosis. These two pathways intersect downstream: RIPK3 can activate caspase-8 to cleave GSDMD, inducing pyroptosis in heatstroke; conversely, when caspase-8 is inhibited, RIPK3 receives LPS signals to activate MLKL, triggering necroptosis in sepsis.

In 2022, Professor Lu Ben’s team (Yuan et al., 2022) discovered that Z-DNA-binding protein 1 (ZBP1) mediates the pathological features of heat stroke by triggering receptor interaction protein kinase 3 (RIPK3)-induced mixed lineage kinase-like protein (MLKL)-dependent cell necroptosis. This finding significantly advanced research on heat stroke. Gasdermin D (GSDMD) can trigger another form of cell death—pyroptosis (Kayagaki et al., 2015). The excessive activation of this cell death pathway is precisely the key mechanism leading to sepsis complicated by DIC or organ failure. The two signaling pathways activate differently, yet their downstream signals converge extensively. As shown in Figure 1.

### 2.1 Differential activation of sensors: ZBP1 perceives heat stress, caspase-11 detects cytoplasmic LPS

During heat stress, heat shock proteins (HSPs) are employed to correct misfolded proteins. Heat shock factor 1 (HSF1), as a member of the heat shock factor family, participates in the transcriptional regulation of numerous heat shock proteins while also regulating cell death during heat stress (Wang et al., 2024). On one hand, heat stress directly induces ZBP1 oligomerization via its RHIM-A domain (Yuan et al., 2022). On the other hand, when heat stress elevates core body temperature to abnormal levels, HSF1 trimerizes, becomes phosphorylated, and translocates to the nucleus. There, it binds to the heat shock element (HSE) within the ZBP1 gene promoter region, recruiting co-activators such as p300 to upregulate ZBP1 transcription. Both pathways activating ZBP1 are independent of Z-nucleic acid sensors (Yuan et al., 2022). When ZBP1 expression is downregulated, the survival rate of heat stroke mice significantly increases; this indicates that ZBP1, acting as a heat stress signal receptor, triggers downstream effector mechanisms.

Sepsis arises from systemic infection, and both human sepsis patients and animal models exhibit apoptosis and activation of cysteine-aspartic acid proteases (caspases). During early infection, cytoplasmic LPS is recognized and bound by the CARD domain of procaspase-11. Subsequently, procaspase-11 oligomerizes and activates caspase-11, triggering subsequent pyroptotic mechanisms. High-mobility group box protein B1 (HMGB1), a key endogenous damage-associated molecular patterns (DAMPs) primarily released by the liver, forms a crucial protein-receptor pair with the receptor for advanced glycation end products (RAGE). During sepsis progression, HMGB1 binds extracellular LPS and is internalized via RAGE endocytosis, activating caspase-11. The incidence and mortality rates of sepsis in caspase-11-deficient mice were significantly lower than those in normal mice (Kang et al., 2002). When HMGB1 is competitively bound, the binding sites for LPS are reduced, leading to diminished activation of caspase-11 and consequently lowering the mortality rate in sepsis (Deng et al., 2018; Tang et al., 2021). Therefore, caspase-11 plays a central monitoring role in sepsis.

ZBP1 and caspase-11 serve as “molecular sentinels” for heats troke and sepsis, respectively. While they recognize distinct signals and activate independent pathways, both trigger severe downstream cell death and inflammatory storm effects.

### 2.2 Death executors: functional similarities, differences, and overlap between MLKL and GSDMD

MLKL, acting as the “executioner” in the cellular necroptosis pathway, possesses an N-terminal 4-helix bundle (4HB) domain responsible for membrane disruption and cell death, linked by a double-helical linker, and a C-terminal pseudo-kinase domain. Under normal conditions, the function of the 4HB domain is inhibited by the C-terminal pseudo-kinase domain. Upon receiving heat stress signals, the RHIM domain of ZBP1 binds to the RHIM domain of RIPK3, forming amyloid fibers through interaction (Koerner et al., 2024). This interaction activates RIPK3. At this point, a conformational change in the C-terminal kinase domain of MLKL allows the 4HB domain to be released and oligomerize. This domain then binds to phospholipids in the cell membrane, localizing to the membrane and forming a membrane pore (Hildebrand et al., 2014). Membrane pores induce Ca^2+^ or Na^+^ influx, leading to leakage of cellular contents and ultimately causing osmotic rupture and cell death (Cai et al., 2014; Chen et al., 2014). Some leaked cellular contents activate the NOD-like receptor family pyrin domain-containing 3 (NLRP3), inducing the self-cleavage and activation of the caspase-1 proenzyme, thereby promoting the maturation and release of IL-1β and IL-18 (Huang et al., 2021). HMGB1 is also released from MLKL pores, which can activate immune cells to trigger the release of additional inflammatory mediators (Baindara et al., 2025). RIPK3 exhibits high specificity for recognizing and phosphorylating MLKL. When mouse MLKL is absent, rat MLKL fails to induce necroptosis in mouse cells (Murphy et al., 2013; Davies et al., 2020; Hildebrand et al., 2014). Under certain conditions, activated MLKL translocates to the lysosomal membrane, where it aggregates into multimers. This process enhances lysosomal membrane permeability (LMP), allowing contents such as cathepsin B (CTSB) to leak into the cytoplasm. These enzymes cleave critical proteins essential for cell survival—including heat shock protein 70 (HSP70) and tubulin—disrupting cellular structure and ultimately leading to cell death (Liu et al., 2024).

GSDMD is a protein composed of a 32 kDa N-terminal domain and a 22 kDa C-terminal domain connected by a peptide linker (Chi et al., 2024). Upon activation by LPS, caspase-11 cleaves human and mouse GSDMD at residues D275 and D276, respectively, releasing the GSDMD N-terminal structure (Shi et al., 2015). The N-terminal structure of GSDMD oligomerizes into a ring-like structure on the plasma membrane, ultimately forming a nanopore (Ding et al., 2016). Numerous cells, such as dendritic cells, macrophages, or certain neutrophils, release large amounts of pro-inflammatory factors like IL-1β, IL-18, and DAMPs through this pathway (Evavold et al., 2018; Heilig et al., 2018). Simultaneously, massive K^+^ efflux occurs within the cell, activating NLRP3 inflammasomes, ultimately triggering severe inflammatory responses and leading to pyroptosis (Banerjee et al., 2018).

The molecular mechanisms of disease rarely operate in isolation; they often converge due to their interconnectedness. Caspase-8 is one of the mediators involved in heat stroke, functioning similarly to caspase-11 (Yuan et al., 2022). Phosphorylated RIPK3 activates caspase-8 to cleave GSDMD, thereby triggering pyroptosis in heat stroke (Yuan et al., 2022). During the progression of sepsis, LPS or tumor necrosis factor-α (TNF-α) can directly activate RIPK3 even when caspase-8 is inhibited. RIPK3 then activates MLKL to induce necroptosis. Concurrently, blocking both RIPK3 and GSDMD significantly improves organ damage in sepsis and reduces mortality (Chen et al., 2020; Wang et al., 2025). These cross-talk signals ultimately converge on the cell death effectors MLKL and GSDMD, further indicating that cell death serves as a common hub linking organ damage caused by both environmental and infectious triggers. Additionally, sepsis is one of the severe complications of heat stroke. When the intestinal barrier is compromised during heat stroke, leading to increased mucosal permeability, enteric endotoxins can enter the bloodstream and trigger sepsis (Lim, 2018). At this point, the ZBP1-RIPK3-MLKL and caspase-11-GSDMD pathways are simultaneously activated and function synergistically, amplifying the cytokine storm and causing further organ damage (Klingensmith and Coopersmith, 2016; Hu et al., 2019). Cytokines released in large quantities after cell rupture or indirectly recruited (He et al., 2015; Kayagaki et al., 2015). These factors activate RIPK3 and caspase-11 in an opposing manner, intensifying the convergence of cell death mechanisms and forming a vicious cycle (Huang et al., 2021; Liao et al., 2023; Man et al., 2017). Some studies suggest that released HMGB1 may serve as a biomarker for heat stroke and sepsis (Baindara et al., 2025; Iba et al., 2025).

## 3 Similar terminal pathological outcomes

### 3.1 Disseminated intravascular coagulation

DIC is characterized by widespread activation of the coagulation cascade, with massive deposition of fibrin within blood vessels leading to extensive thrombus formation in microvasculature (Levi and Ten Cate, 1999). Once platelets and coagulation proteins are depleted, this triggers systemic hemorrhage accompanied by circulatory failure (Levi and Ten Cate, 1999).

In a retrospective observational study examining serum biochemical indicators in 18 patients with heat stroke, elevated levels of whole blood tissue factor (TF) and thrombin-antithrombin complex (TAT) activity were observed. Concurrently, decreased levels of antithrombin, protein S, and protein C were noted. Furthermore, five patients had already developed significant DIC (Huisse et al., 2008). TF is a key substance that triggers the coagulation cascade. During the development of heat stroke, capillary endothelial cell damage releases TF (Wu et al., 2024). Under the stimulation of inflammatory mediators, TF interacts with coagulation factor FVII/FVIIa to trigger the coagulation cascade. This process most likely occurs during the early stages of heat stress or prior to organ dysfunction associated with heat stroke (Proctor et al., 2020; Pawlinski et al., 2010).

The mortality rate increases significantly when sepsis is complicated by DIC (Gando et al., 2013). Following massive exogenous LPS exposure, the liver exhibits increased thrombin generation within microvessels, platelet aggregation, fibrin deposition, and microcirculatory dysfunction. This phenomenon is mitigated or even eliminated by the absence of caspase-11 or GSDMD (Yang et al., 2019). The membrane pores formed at the GSDMD-N-terminal region mediate calcium ion influx and activate transmembrane protein 16 F (TMEM16F), leading to the exposure of phosphatidylserine—originally confined to the inner leaflet of the plasma membrane. Phosphatidylserine enhances TF activity, thereby triggering a potent procoagulant response (Yang et al., 2019; Chen, 2013; Grover and Mackman, 2018).

### 3.2 Multiple organ dysfunction

In the late stages of heat stroke or sepsis, multiple organs throughout the body are affected, including the liver, intestines, kidneys, and brain. Severe cases may progress to multiple organ dysfunction syndrome (MODS), which is one of the primary factors contributing to the poor prognosis and high mortality rates associated with both conditions (Epstein and Yanovich, 2019; Bouchama et al., 2022; van der Poll, 2013).

Heat stroke can cause acute liver injury or liver failure, and some patients may even require a liver transplant to save their lives (Ichai et al., 2019). The lungs are the organs responsible for supplying oxygen to the body. Heat stress leads to increased expression of inflammatory factors such as IL-1β and TNF-α, which recruit neutrophils, macrophages, and monocytes to infiltrate the tissue. This damages the alveolar-capillary barrier, resulting in acute lung injury and subsequently causing hypoxia-induced damage to other organs (Liu et al., 2020c). Exposure to high temperatures causes rhabdomyolysis, resulting in severe damage to the kidneys (Zhang et al., 2025). High temperatures can also directly damage the fragile intestinal barrier, leading to bacterial translocation (Ma et al., 2025). The central nervous system experiences dysfunction under high-temperature stress, leading to conditions such as coma and delirium (Yang et al., 2025).

The liver plays a crucial role in the progression of sepsis by eliminating invading pathogens, regulating the production of inflammatory factors, and maintaining immune homeostasis (Strnad et al., 2017). However, it also becomes one of the organs most severely affected by sepsis. Under LPS stimulation, elevated alanine aminotransferase (ALT) and aspartate aminotransferase (AST) levels in the liver indicate significant damage (Liu et al., 2020a). Peripheral blood mononuclear cells from sepsis patients exhibit greater GSDMD cleavage than those from healthy individuals. Moderate pyroptosis facilitates pathogen clearance, but excessive pyroptosis damages lung tissue, inducing acute lung injury that leads to pulmonary dysfunction or acute respiratory distress syndrome (ARDS) (Font et al., 2020; Confalonieri et al., 2017; Zhao et al., 2024; Zheng et al., 2021; Li et al., 2025). Under the combined effects of inflammatory stimulation, coagulation dysfunction, microcirculatory impairment, and hemodynamic alterations caused by sepsis, the kidneys may sustain acute injury or failure (Molema et al., 2022). Central nervous system injury secondary to sepsis-related systemic infection occurs when the blood-brain barrier is compromised and uncontrolled inflammatory responses stimulate the nervous system, triggering a systemic inflammatory storm that impairs brain function (Gofton and Young, 2012; Meneses et al., 2019).

The essence of the body’s response in heat stroke lies in the failure of thermoregulation, while that in sepsis stems from uncontrolled infection and inflammatory storms. However, DIC and MODS represent the common causes of death in both heat stroke and sepsis patients. This phenomenon suggests that the convergence point between these two diseases may occur during the execution phase of cell death. Blocking this “convergence point” is key to preventing the onset of DIC and MODS.

## 4 Therapeutic strategies: from traditional limitations to targeted approaches

### 4.1 Limitations of existing therapies

Heat stroke and sepsis are both acute critical illnesses, and timely, effective treatment is crucial for saving patients’ lives. Due to the rapid progression of the condition, clinical interventions targeting only the underlying cause often yield limited benefits. The primary treatment strategy for heat stroke currently involves rapidly lowering the patient’s body temperature. Common methods include cold water immersion and intravenous infusion of pre-chilled saline (Demartini et al., 2015; Zhang et al., 2024). However, this strategy is ineffective in certain situations. If the body is exposed to high temperatures for an extended period, harmful mechanisms have already been triggered, and cooling cannot control the subsequent inflammatory storm (Bouchama et al., 2022; Barletta et al., 2025).

The use of antibiotics and organ support therapies, coupled with the advancement of intensive care medicine, has significantly reduced mortality rates among sepsis patients. However, substantial challenges remain in treating sepsis. Even when patients survive, they often face poor quality of life due to various sequelae (Giamarellos-Bourboulis et al., 2024). The delayed use of antibiotics in clinical settings correlates positively with mortality rates in sepsis patients. Long-term misuse of antibiotics fosters drug resistance, rendering antibiotics ineffective or leading to secondary infections. Consequently, antibiotic therapy for sepsis remains fraught with numerous challenges and shortcomings (de la Fuente-Nunez et al., 2023).

### 4.2 Novel strategies targeting death pathways

For heat stroke and sepsis, effective treatments should not only reduce mortality risk but also improve prognosis and enhance patients’ quality of life. Targeting inhibition of molecular receptors ZBP1 or caspase-11 activation may better block disease progression, but currently few inhibitors exist for ZBP1 and caspase-11. Dr. R. Huang (Huang et al., 2025) developed a covalent recognition-based targeted protein degradation chimera (C-PROTAC) capable of specifically degrading ZBP1. C-PROTAC contains a ZBP1 ligand that specifically binds to ZBP1, while its E3 ubiquitin ligase ligand promotes ZBP1 ubiquitination and degradation of the ZBP1-PROTAC complex. *In vitro* experiments demonstrate that C-PROTAC effectively degrades ZBP1 during viral infection, thereby enhancing cell survival rates (Huang et al., 2025). But research on C-PROTACs remains limited and has only demonstrated efficacy *in vitro* cellular models. Whether the same pharmacological effects can be reproduced under complex *in vivo* physiological conditions remains unclear. Baicalin, a flavonoid compound, exhibits a dose-dependent inhibition of macrophage caspase-11 activation *in vitro* experiments (Ye et al., 2021). Blocking the transmission of upstream signals represents the fastest and most direct method to inhibit the activation of downstream death pathways. However, for these two diseases, a narrow window for early intervention stands as one of their most prominent clinical characteristics. Currently, there remains a lack of biomarkers capable of rapidly and accurately assessing disease progression. Therefore, relying solely on inhibiting upstream targets is clearly insufficient for ensuring safety.

RIPK3 has been identified as a potential drug target (Newton et al., 2014). GSK872 is a classic RIPK3 inhibitor that suppresses TLR3- induced necroptosis and improves outcomes in glaucoma and Parkinson’s disease (Kaiser et al., 2013; Mandal et al., 2014; Liu et al., 2022; Park et al., 2024). The small molecule Zharp-90 can block RIPK3 activation in humans and in both mouse and rat models, thereby improving TNF-α induced systemic inflammatory response syndrome (SIRS). Additionally, Zharp-90 exhibits favorable *in vitro* safety and *in vivo* pharmacokinetic parameters (Mandal et al., 2014; Xia et al., 2020). A small-molecule compound salt-induced kinase inhibitor, HG-9-91-01, directly suppresses RIPK3 kinase activity, blocks TNF-α or TLR-induced necroptosis, and has been demonstrated in animal studies to inhibit *staphylococcus* aureus-induced lung injury (Huang et al., 2022). Chuan-hui Xu (Xu et al., 2022) found that AZD5423 improves acute kidney injury induced by ischemia-reperfusion or cisplatin by specifically inhibiting RIPK3 kinase activity. N-deethylsulfamone (NSA) is a widely studied MLKL inhibitor. In neuroinflammation induced by LPS or poly (I:C), NSA exerts its effects by reducing the production of inflammatory cytokines, HMGB1, DAMPs, and reactive oxygen species (ROS), as well as suppressing p-MLKL expression and inhibiting the activation of the necroptosis signaling pathway. Additionally, NSA improves colitis (Kim et al., 2025; Yang et al., 2022). Disulfiram is a medication approved by the U.S. Food and Drug Administration (FDA) for treating alcohol dependence. It has been discovered that disulfiram can also block the formation of GSDMD pores, thereby reducing the release of inflammatory factors and inhibiting the occurrence of pyroptosis (Hu et al., 2020). Dimethylformamide (DMF) can react with GSDMD to form S-(2-succinyl)-cysteine, thereby limiting its interaction with caspase-11 and achieving the goal of inhibiting pyroptosis (Humphries et al., 2020). GI-Y1 is also a GSDMD inhibitor with a mechanism of action similar to disulfiram, capable of preventing GSDMD pore formation and inhibiting the occurrence of pyroptosis (Zhong et al., 2023).

The efficacy of most of the aforementioned target inhibitors has been validated in animal or cellular experiments, with some even being applied in clinical settings. They all hold promise for future development into drugs for treating heat stroke or sepsis. Unfortunately, research on these inhibitors still faces certain limitations and shortcomings. Currently, few studies directly test these target inhibitors in heat stroke or sepsis models, instead extrapolating their efficacy from other disease models. Furthermore, these basic experimental designs are conducted under relatively simple and idealized conditions. They do not address critical questions such as whether the drugs exhibit dose dependency, the extent of their safe dosage range, their bioavailability, whether different species exhibit comparable sensitivity, or whether their use might inhibit the functions of other normal proteins.

## 5 Future direction: broad-spectrum interventions at the mechanism intersection

**FIGURE 2:**
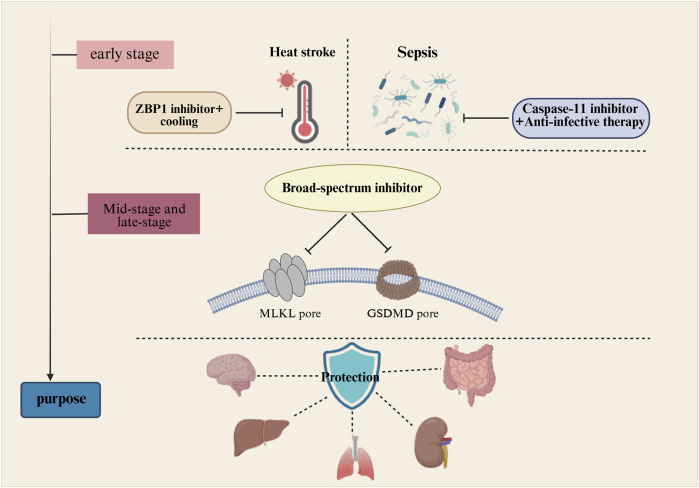
Therapeutic strategy for heat stroke and sepsis: Early specific intervention and late broad-spectrum suppression. Early-stage interventions target molecular sensors and disease triggers specific to heatstroke and sepsis, aiming to block disease signal transmission at its source. Late-stage interventions employ broad-spectrum inhibitors to simultaneously block both necrotic apoptosis and pyroptosis, thereby protecting vital organs.

Heat stroke and sepsis exhibit distinct characteristics in their early stages but share similarities in their late progression. Therefore, interventions must be tailored to the specific developmental phase of each condition. As shown in Figure 2.

In the initial phase, disease progression can be controlled by separately blocking their external triggers and the activation of molecular sensors: rapidly cooling combined with ZBP1 inhibitors halts heat stroke progression, while anti-infective therapy paired with caspase-11 inhibitors interrupts sepsis progression. As the disease progresses into the mid-to-late stages, the harmful mechanisms of both conditions may converge, necessitating combined blockade. A broad-spectrum inhibitor must be developed to simultaneously suppress the activation of targets such as RIPK3, MLKL, and GSDMD, thereby maximally limiting cellular death. In cases of heat stroke complicated by sepsis, concurrent anti-infective therapy and broad-spectrum targeted inhibition are required. When coagulation dysfunction occurs, heparin administration may be added to the treatment regimen (Tang et al., 2021; Wang et al., 2014; Perchick et al., 1975; Iba et al., 2024). When abnormalities are detected in organ injury markers such as AST, ALT, creatinine, or blood urea nitrogen, organ support therapies such as continuous renal replacement therapy and fluid resuscitation should be initiated (Wang et al., 2022; Liu et al., 2020b). Pathological mechanisms such as mitochondrial damage, oxidative stress, and ferroptosis also play a contributory role in the progression of heat stroke and sepsis (Jin et al., 2018; Fang et al., 2024). Although not elaborated in detail in this review, these approaches can be integrated organically. When implementing early-stage specific interventions for heat stroke and sepsis alongside late-stage broad-spectrum target inhibition, it is essential to concurrently consider protecting cellular mitochondrial function and scavenging reactive oxygen species. This comprehensive strategy maximizes the mitigation of disease-induced damage to the body.

## 6 Current limitations in understanding and challenges in clinical translation

Although the roles of individual targets within the ZBP1-RIPK3-MLKL axis and the caspase-11-GSDMD axis in heat stroke and sepsis have been relatively well defined, current research still faces significant limitations. Most studies rely solely on animal or cellular experiments, making it difficult to directly extrapolate and apply findings to humans. Furthermore, few reports exist on the use of signaling pathway target inhibitors for treating heat stroke or sepsis. The convergence of cellular death mechanisms in these diseases remains superficially understood, lacking in-depth analysis of molecular switching mechanisms. As a complex system, the body exhibits varying pathway dependencies across tissues and organs, leading to differing sensitivities to target inhibitors. The narrow therapeutic window for disease treatment, the disconnect between basic research and clinical practice, and the lack of key disease biomarkers all pose significant challenges. Converting inhibitors into clinical drugs requires substantial funding and lengthy clinical trials to validate their safety and efficacy. Consequently, translating these discoveries into actual clinical treatment strategies remains a major hurdle. Moving forward, we must prioritize driving substantive progress from theoretical frameworks through fundamental research to clinical applications.

## 7 Conclusion

Although heat stroke and sepsis originate from different disease triggers, they converge toward the same pathological outcome. By comparing and integrating their mechanisms of programmed cell death, this study reveals pathway convergence during disease progression. This suggests that treatments targeting only the cause or a single target have limited efficacy. Future therapeutic strategies should consider specific early-stage molecular signaling blockade and synergistic inhibition of late-stage death pathways for both diseases. Developing broad-spectrum inhibitors capable of simultaneously suppressing both necroptosis and pyroptosis is crucial for reducing mortality and improving outcomes in both heat stroke and sepsis.

## Data Availability

The original contributions presented in the study are included in the article/supplementary material, further inquiries can be directed to the corresponding authors.
